# Modulation of Cardiac Ryanodine Receptor Channels by Alkaline Earth Cations

**DOI:** 10.1371/journal.pone.0026693

**Published:** 2011-10-21

**Authors:** Paula L. Diaz-Sylvester, Maura Porta, Julio A. Copello

**Affiliations:** 1 Department of Pharmacology, Southern Illinois School of Medicine, Springfield, Illinois, United States of America; 2 Department of Physiology, Midwestern University, Downers Grove, Illinois, United States of America; Georgia State University, United States of America

## Abstract

Cardiac ryanodine receptor (RyR2) function is modulated by Ca^2+^ and Mg^2+^. To better characterize Ca^2+^ and Mg^2+^ binding sites involved in RyR2 regulation, the effects of cytosolic and luminal earth alkaline divalent cations (M^2+^: Mg^2+^, Ca^2+^, Sr^2+^, Ba^2+^) were studied on RyR2 from pig ventricle reconstituted in bilayers. RyR2 were activated by M^2+^ binding to high affinity activating sites at the cytosolic channel surface, specific for Ca^2+^ or Sr^2+^. This activation was interfered by Mg^2+^ and Ba^2+^ acting at low affinity M^2+^-unspecific binding sites. When testing the effects of luminal M^2+^ as current carriers, all M^2+^ increased maximal RyR2 open probability (compared to Cs^+^), suggesting the existence of low affinity activating M^2+^-unspecific sites at the luminal surface. Responses to M^2+^ vary from channel to channel (heterogeneity). However, with luminal Ba^2+^or Mg^2+^, RyR2 were less sensitive to cytosolic Ca^2+^ and caffeine-mediated activation, openings were shorter and voltage-dependence was more marked (compared to RyR2 with luminal Ca^2+^or Sr^2+^). Kinetics of RyR2 with mixtures of luminal Ba^2+^/Ca^2+^ and additive action of luminal plus cytosolic Ba^2+^ or Mg^2+^ suggest luminal M^2+^ differentially act on luminal sites rather than accessing cytosolic sites through the pore. This suggests the presence of additional luminal activating Ca^2+^/Sr^2+^-specific sites, which stabilize high P_o_ mode (less voltage-dependent) and increase RyR2 sensitivity to cytosolic Ca^2+^ activation. In summary, RyR2 luminal and cytosolic surfaces have at least two sets of M^2+^ binding sites (specific for Ca^2+^ and unspecific for Ca^2+^/Mg^2+^) that dynamically modulate channel activity and gating status, depending on SR voltage.

## Introduction

During excitation-contraction coupling in the heart, calcium ions (Ca^2+^) are mobilized from the sarcoplasmic reticulum (SR) to the cytosol through ryanodine receptor Ca^2+^ release channels (RyR isoform 2, RyR2), located at the terminal cisternae of the SR [1], [2], [3], [4]. Previous research has shown that this massive intracellular Ca^2+^ release in cardiac muscle depends on extracellular Ca^2+^ entry through the L-type Ca^2+^ channels (reviewed in [5], [6]). The process was termed "calcium induced calcium release". Accordingly, it has also been shown that isolated RyR2 are Ca^2+^-gated channels [6], [7], [8], [9].

RyR2 display a biphasic response to cytosolic Ca^2+^: 10–100 µM Ca^2+^ induces maximal activation, whereas 1–10 mM Ca^2+^ is inhibitory [1], [2], [3], [4], [10]. This suggests the existence of two different types of cytosolic Ca^2+^ binding sites: activating sites with high affinity (micromolar) and inhibitory sites with low affinity (millimolar). RyR2 are also sensitive to cytosolic Mg^2+^ [11], [12]. However, the effect of Mg^2+^ is inhibitory. It is thought that Mg^2+^ inhibition of RyR2 function involves both competition of Mg^2+^ with Ca^2+^ binding to cytosolic activating sites and Mg^2+^ binding to additional inhibitory cytosolic Mg^2+^ binding sites [11], [12]. Interference of Ba^2+^ with cytosolic Ca^2+^-mediated activation of RyR2 has also been reported, although the presence of one or multiple binding sites has not been elucidated [13].

Current evidence supports the existence of additional binding sites for alkaline earth divalent ions (M^2+^) at the luminal surface of the RyR2 [13], [14]. Affinity to luminal Ca^2+^ has previously been measured for ATP-activated RyR2 and the reported values range from ∼50 µM [15] to millimolar levels [14], [16]. The inhibitory effect of luminal Mg^2+^ on Ca^2+^-activated [17] and ATP-activated RyR2 has also been reported [15]. The mechanism of action of luminal M^2+^ is still unclear although a combination of luminal M^2+^ effects on cytosolic Ca^2+^ and ATP modulation and the “trans effect” of lumen-to-cytosol M^2+^ flux acting on cytosolic M^2+^ sites of single RyR2 has been proposed to play a role [4], [17], [18], [19], [20].

The aim of this work was to gain new insights on how different binding sites for M^2+^ ions, both in the lumen and the cytosolic surfaces of the RyR2, affect the gating characteristics of channels reconstituted into planar lipid bilayers. Experiments were also conducted to determine if the flux of different divalent cations through the channel plays a role in RyR2 modulation.

The data presented here suggest that RyR2 channel behavior can be modified by M^2+^ interaction with cytosolic Ca^2+^-specific and M^2+^-unspecific sites (which under physiological conditions would bind Mg^2+^ and Ca^2+^). Moreover, the binding of M^2+^ to luminal sites differentially affected RyR2 gating kinetics and voltage-dependence as well as RyR2 sensitivity to cytosolic Ca^2+^ and cytosolic caffeine. Some of the results have been presented in a preliminary form [21], [22].

## Methods

### Drugs and chemicals

CaCl_2_ standard for calibration was from Word Precision Instruments Inc (Sarasota, FL). Phospholipids were obtained from Avanti (Alabaster, AL), and decane from Aldrich (Milwaukee, WI). BAPTA (1,2-bis (o-aminophenoxy) ethane-N,N,N',N'-tetraacetic acid), dibromoBAPTA (1,2-bis 2-bis(o-amino-5-bromophenoxy)ethane-N,N,N',N'-tetraacetic acid), Ba(OH)_2_, Ca(OH)_2_, Mg(OH)_2_, Sr(OH)_2,_ CsOH, CsCl, and HEPES were obtained from Fluka (Boca Raton, Fl). All other drugs and chemicals were from Sigma or were reagent grade.

### Sarcoplasmic reticulum microsomes

All procedures with animals were designed to minimize pain and suffering and conformed to the guidelines of the National Institutes of Health. SIUMED animal research procedures have AAALAC accreditation and PHS assurances numbers 000551 and A3209-01 respectively. The committee on the Use and Care of Laboratory Animals of Southern Illinois University School of Medicine reviewed and approved the protocols for animal use in our laboratory (196-05-021 and 196-08-003). Sarcoplasmic reticulum (SR) microsomes were obtained from pig heart ventricle using heart homogenization and ultracentrifugation steps that follow the procedures published by Chamberlain et al. [23]. SR pellets obtained after high speed centrifugation were resuspended in 290 mM sucrose - 5 mM Imidazole buffer (pH = 7), aliquoted in cryovials (300 µl each) and kept in liquid nitrogen (better and safer long-term storage). Every month, a few cryovials were used to generate smaller aliquots of membranes (15 µl each) which were stored at –80°C for easy access. For experiments, aliquots were quickly thawed in water, kept on ice and used within 3–5 hours.

### Bilayer technique

Reconstitution of RyR2 in planar lipid bilayers was performed as previously described [10]. Briefly, planar lipid bilayers were formed on 80 to 100 µm-diameter circular holes in teflon septa, separating two 1.3 ml compartments. The *trans* compartment was filled with HEPES-M^2+^ solution containing HEPES 250 mM and M(OH)_2_ 53 mM, pH 7.4 (M^2+^ was either Mg^2+^, Ca^2+^, Sr^2+^ or Ba^2+^). The *trans* compartment was clamped at 0 mV using an Axopatch 200B patch-clamp amplifier (Axon Instruments, Foster City, CA). The *cis* compartment (ground) was filled with HEPES-Tris solution containing HEPES 250 mM and TrisOH 118 mM, pH 7.4. Bilayers of a 5∶4∶1 mixture of bovine brain phosphatidylethanolamine, phosphatidylserine and phosphatidylcholine (45–50 mg/ml in decane) were painted onto the holes of teflon septa from the *cis* side. Sarcoplasmic reticulum microsomes (5–15 µg) were then added to the *cis* solution followed by 500–1000 mM CsCl and 1 mM CaCl_2_ to promote vesicle fusion. After RyR currents (or Cl^−^ currents >100 pA at 0 mV) were observed, the *cis* chamber was perfused with HEPES-TRIS solution for 5 min at 4 ml/min. A mixture of BAPTA and dibromo-BAPTA was used to buffer free [Ca^2+^] on the cytosolic surface of the channel ([Ca^2+^]_cyt_) [10]. As previously done [10], RyR channels were identified by current amplitudes (∼3.5 pA at 0 mV), slope conductance (∼100 pS), reversal potential (∼−45 mV, *trans* - *cis*) and response to diagnostic ligands (e.g., ryanodine, Ca^2+^, ATP, caffeine and Ruthenium Red). RyR channel currents are depicted as positive (upward deflections of the current) in figures and reflect cation flux from the *trans* (luminal) to the *cis* (cytosolic) compartment. Membrane voltages always represent the difference between *trans - cis* compartments (in mV).

### Single channel analysis

Channel currents were first filtered through the Axopatch 200B low-pass Bessel filter at 2 kHz, digitized at 20 kHz with an analog to digital converter (Digidata 1320, Axon Instruments) and stored on DVD. Recordings were analyzed using pClamp9 software (Axon Instruments). Analysis with this program included open times, closed times and open probabilities (P_o_), which were determined by half-amplitude threshold analysis of single-channel recordings as done before [10]. In multichannel experiments, the global open probability (nP_o_) was estimated. In the figures we show the P_o_ (for single channels) or nP_o_/x (for multiple channels, with x representing the maximal number of current levels observed).

### Statistical Analysis

Data are shown as means ± S.E.M. of n measurements. Statistical comparisons between groups were performed with Student's t test for paired samples. Differences were considered statistically significant at P<0.05.

## Results

In this work, we studied the modulation of RyR2 by alkaline earth cations (M^2+^: Mg^2+^, Ca^2+^, Sr^2+^ or Ba^2+^) added either to the cytosolic or luminal channel surface. In all cases, we measured the activity of RyR2 (from pig heart ventricular microsomes) reconstituted in planar lipid bilayers. Unless explicitly stated, recordings were done at 0 mV (transmembrane voltage).

### At least two different types of M^2+^ binding sites exist on RyR2 cytosolic surface

**Figure 1:**
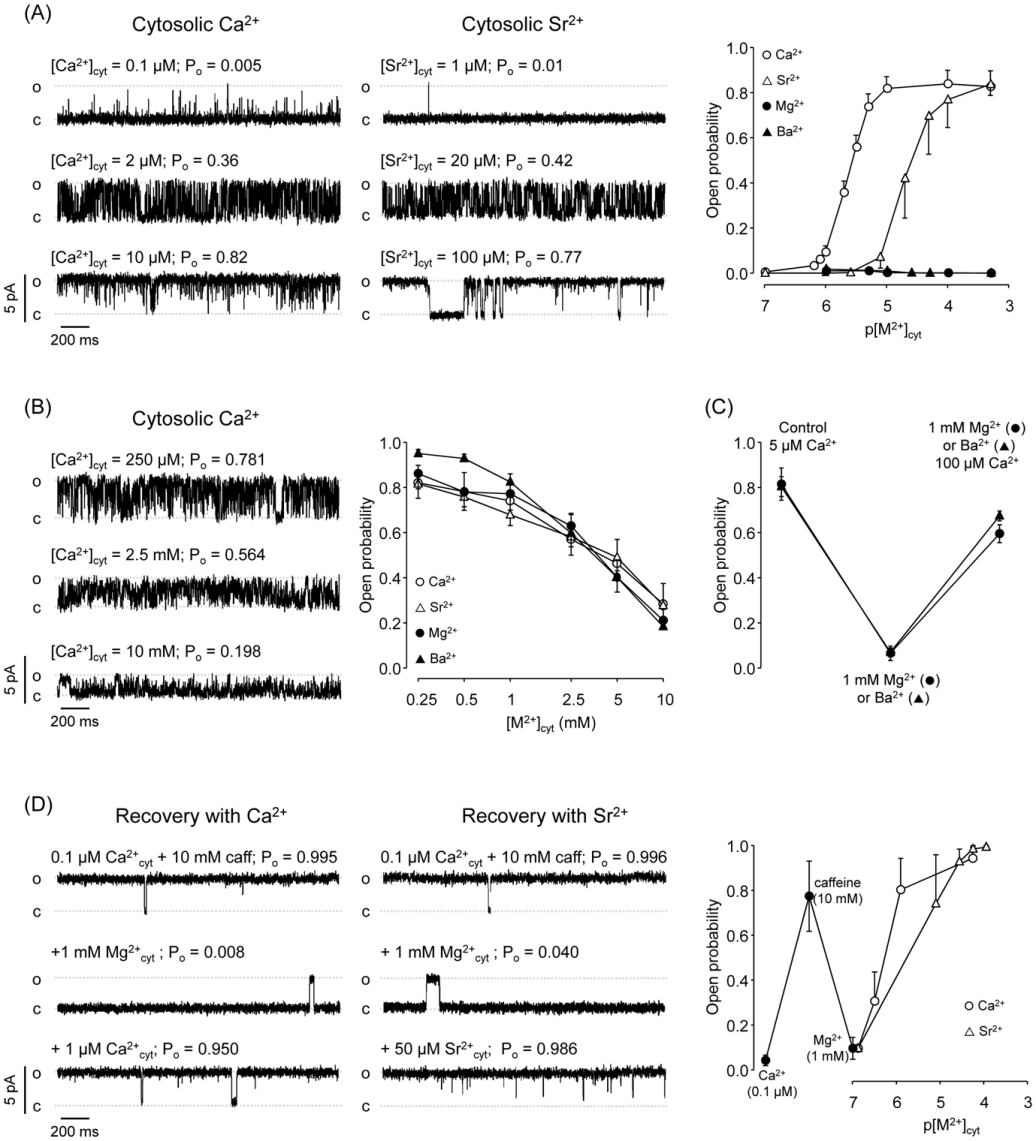
Effects of M^**2+**^ added to the cytosolic surface of RyR2. (A) *RyR2 are activated by micromolar levels of cytosolic Ca^2+^/Sr^2+^ but not Mg^2+^/Ba^2+^*. Representative RyR2 recordings for channels activated by Ca^2+^ (left panel) or Sr^2+^ (middle panel) added to the cytosolic compartment. All recordings were made at a holding potential (V_m_) of 0 mV with luminal Ca^2+^ (50 mM) as the current carrier. The right panel shows mean open probability (P_o_) of RyR2 channels as a function of free earth alkaline divalent cation concentration [M^2+^] varying from 100 nM to 500 µM. P_o_'s are mean values from n = 10 (Ca^2+^, open circles), 6 (Sr^2+^, open triangles), and 5 experiments (Mg^2+^ and Ba^2+^, filled circles and triangles respectively). Values are shown as mean ± SEM. Ca^2+^ activated the channels with an EC_50_ = 2.3±0.1 µM and n_H_ = 2.4±0.1. With Sr^2+^ EC_50_ = 20.2±1.0 µM and n_H_ = 2.2±0.2. (B) *RyR2 are inhibited by high (mM) concentrations of M^2+^*. Left panels show representative recordings of RyR2 fully activated by cytosolic Ca^2+^ (100 µM) which were exposed to cumulative doses of Ca^2+^ added to the cytosolic side of channel. The Mean P_o_ of RyR2 as a function of [M^2+^] varying from 0.25 to 10 mM is shown in the right panel. Again, luminal Ca^2+^ (50 mM) was current carrier and V_m_ = 0 mV. All M^2+^ tested had similar inhibitory action when applied at millimolar concentrations. IC_50_'s were: 3.5±1.3; 4.5±0.4; 5.6±0.7 and 5.5±0.3 mM (Ba^2+^, Mg^2+^, Sr^2+^ and Ca^2+^ respectively). (C) *Cytosolic Mg^2+^ and Ba^2+^ interfered Ca^2+^activation of RyR2*. Mean P_o_ of RyR2 at 5 µM free cytosolic Ca^2+^ was inhibited by addition of 1 mM Mg^2+^ (circles) or 1 mM Ba^2+^ (Triangles) (n = 5 each). Subsequent increase of cytosolic Ca^2+^ to 100 µM counteracted the inhibition by Mg^2+^ or Ba^2+^. (D) *Ca^2+^ and Sr^2+^ counteract inhibition by Mg^2+^ of caffeine-activated RyR*. RyR2 studies were performed at V_m_ = 0 mV with luminal Ca^2+^ (50 mM) as current carrier. In the presence of 0.1 µM free cytosolic Ca^2+^, RyR2 are fully activated by 10 mM caffeine (top traces). 1 mM cytosolic Mg^2+^ inhibited RyR2 (middle traces). Increasing cytosolic Ca^2+^ 1 µM (bottom left trace) or Sr^2+^ to ∼20 µM (bottom right trace) recovered RyR2 activity. The right panel shows that mean P_o_ of RyR2 at 0.1 µM cytosolic Ca^2+^ was greatly enhanced by adding caffeine (10 mM). Addition of 1 mM Mg^2+^ inhibited the channels. Subsequent increase in cytosolic Ca^2+^ levels to >1 µM or Sr^2+^ to 5–50 µM recovered channel activity (n = 5).

Figure 1A shows representative recordings of RyR2 channels activated by Ca^2+^ (left panel) or Sr^2+^ (middle panel) added to the cytosolic compartment. All recordings were made at a holding potential (V_m_) of 0 mV with luminal Ca^2+^ (50 mM) as current carrier. As previously reported (reviewed in [1], [2], [3], [4]), the channels activated when cytosolic Ca^2+^ increased to micromolar levels. Figure 1A, right panel summarizes open probability (P_o_) data from n = 10 RyR2 experiments (open circles). From these experiments, we estimated that the effective concentration of Ca^2+^ that induces half maximal P_o_ (EC_50_) was 2.3±0.1 µM. Channel activation had a Hill coefficient (n_H_) of 2.4±0.1. RyR2 were also activated with increasing Sr^2+^ levels, as shown in the recordings (Fig. 1A , middle panel) and in the summary of n = 6 experiments (Fig. 1A, right panel, open triangles). However, EC_50_ for Sr^2+^ was 20.2±1.0 µM (∼10 times higher than EC_50_ for Ca^2+^). Similar as with Ca^2+^, n_H_ with Sr^2+^ was 2.2±0.2. These n_H_ >1 suggest that multiple interacting M^2+^ binding sites specific for Ca^2+^>Sr^2+^ are involved in Ca^2+^ or Sr^2+^-induced RyR2 activation. As shown in Fig. 1A (right panel, filled circles and triangles), RyR2 did not activate when cytosolic Mg^2+^ or Ba^2+^ levels were increased (from 0.1 to 500 µM). This confirms that cytosolic M^2+^ activating sites are selective for Ca^2+^ and Sr^2+^.

It is well known that RyR2 response to cytosolic Ca^2+^ is biphasic. Micromolar Ca^2+^ levels activate RyR2 while millimolar Ca^2+^ levels inhibit the channel ([10] reviewed in [2], [5]). Yet, the RyR2 response to millimolar Ca^2+^ is heterogeneous, as only a fraction of the channels would be significantly inhibited by 5 mM Ca^2+^ [10]. This population of sensitive RyR2 was used to examine the M^2+^ selectivity of the cytosolic low affinity inhibitory sites. For that, we first exposed RyR2 to 200 µM cytosolic Ca^2+^ (to elevate P_o_ to a peak level) and subsequently, added cumulative doses of either Ca^2+^, Sr^2+^, Ba^2+^ or Mg^2+^. As shown in Fig. 1B, all the M^2+^ tested had a similar inhibitory action on RyR2 P_o_. The concentrations for half maximal inhibition (IC_50_) were also similar, ranging from 3.5 to 5.7 mM (see Fig. 1B, legend).

We found that Mg^2+^ and Ba^2+^ interfere with Ca^2+^/Sr^2+^-induced activation of RyR2. Figure 1C shows that in the presence of 5 µM cytosolic Ca^2+^ RyR2 activated, reaching a high P_o_ value. Subsequent addition of either 1 mM Ba^2+^ or 1 mM Mg^2+^. reduced P_o_ by 90.5±2.8% and 92.7±4.2% respectively. The inhibition induced by cytosolic Mg^2+^ or Ba^2+^ was counteracted by further increasing cytosolic Ca^2+^ to 100 µM (Fig. 1 C).

It is known that caffeine increases RyR2 sensitivity to cytosolic Ca^2+^ activation [2], [4], [24]. In Fig. 1D we show that in the presence of 10 mM caffeine, only 100 nM cytosolic Ca^2+^ is required to reach a high P_o_ level (comparable to that in Fig. 1C, with 5 µM cytosolic Ca^2+^). Still, the Mg^2+^ concentrations required to induce equivalent degrees of inhibition are the same, regardless of the presence/absence of caffeine. However, much less Ca^2+^ was required to counteract the effect of Mg^2+^ (1 µM versus 50–100 µM Ca^2+^ in the absence of caffeine; compare Fig. 1C and 1D). Our current and previous results [10], [11], [24] indicate that caffeine increases RyR2 sensitivity to cytosolic Sr^2+^ and Ca^2+^ activation by ∼20–50 times but it does not significantly increase RyR2 sensitivity to Mg^2+^ and Ba^2+^ inhibition.

### Luminal M^2+^ affect the response of RyR2 to Ca^2+^ and caffeine

**Figure 2:**
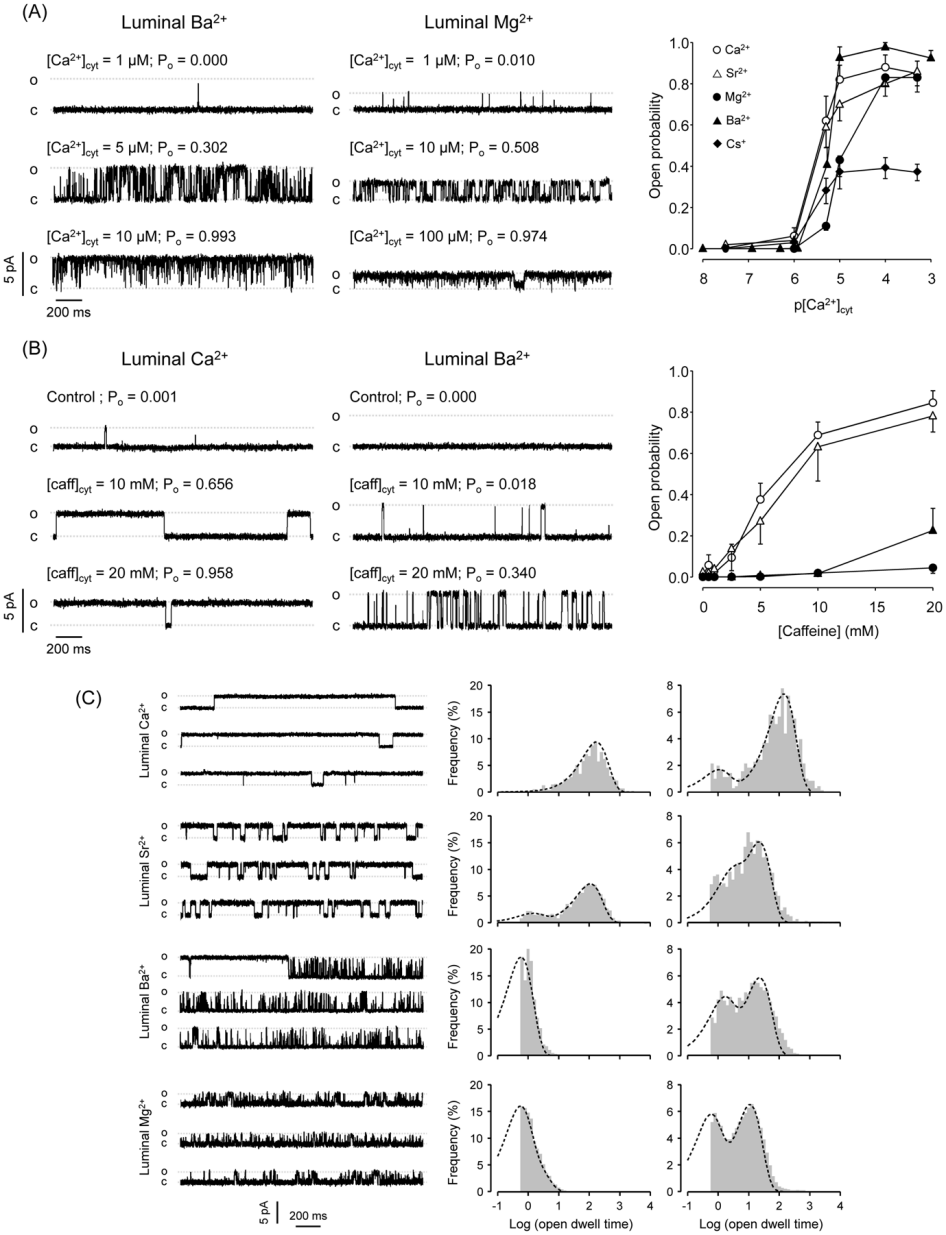
Effects of M^**2+**^ added to the luminal surface of RyR2. (A) *Luminal M^2+^ alters open probability of Ca^2+^-activated RyR2*. Representative recordings of RyR exposed to increasing concentration of cytosolic Ca^2+^, in the presence of luminal Ba^2+^ (left panel) or Mg^2+^ (middle panel). Mean P_o_ as a function of cytosolic Ca^2+^ observed in RyR2 bathed with different luminal cations (right panel). Maximal open probability values of channels exposed to 50 mM luminal M^2+^ were higher than those observed with 100 mM luminal Cs^+^ (p<0.05). The EC_50_ for cytosolic Ca^2+^ activation in the presence of luminal Mg^2+^ was 10.4±0.6 µM (n = 4). This is substantially higher than the EC_50_ with luminal Cs^+^, Ca^2+^ or Sr^2+^ (3.2±0.3 µM, n = 5; 2.9±0.3, n = 7; and 3.4±0.4 µM, n = 5 respectively). EC_50_ with Ba^2+^ was intermediate (6.7±0.3 µM, n = 7). (B) *Luminal M^2+^ effect is more evident in the presence of caffeine.* Representative recordings of RyR exposed to increasing concentration of cytosolic caffeine, in the presence of luminal Ca^2+^ (left panel) or Ba^2+^ (middle panel). The left panel shows mean P_o_ as a function of cytosolic caffeine observed in RyR2 bathed with different luminal M^2+^ (n = 5 in each condition). Recordings were made at 100 nM cytosolic free Ca^2+^. In the presence of luminal Ca^2+^ or Sr^2+^, caffeine activated RyR2 to high P_o_ (EC_50_'s were 5.8±0.5 and 6.4±0.4 mM respectively). With luminal Ba^2+^ or Mg^2+^, RyR2 poorly activated or remained closed. (C) *Luminal M^2+^ affects the gating of caffeine-activated RyR2*. Left panels: representative single channel recordings. Right panels: Open and closed dwell-time distribution histograms of caffeine-activated RyR2 bathed with different luminal M^2+^. All recordings were made at a holding potential of 0 mV. The cytosolic solutions contained 100 nM cytosolic Ca^2+^ and 20 mM caffeine. Dwell open (τ^o^) and closed (τ^c^) times with different luminal M^2+^ were obtained by fitting the logarithmic dwell time distributions (open or close events distributions) with two components. Openings with luminal Ca^2+^ distributed with τ^o^ = 154 ± 1 ms (100% events) while closures distributed with τ^c^_1_ = 1.00 ± 0.21 ms (17% of the events) and τ^c^_2_ = 143 ± 9 ms (83%). Values with Sr^2+^ were τ^o^_1_ = 1.32 ± 0.14 ms (18%), τ^o^_2_ = 114 ± 5 ms (82%), τ^c^_1_ = 2.2 ± 0.3 ms (28%) and τ^c^_2_ = 23.0 ± 2.5 ms (72%). Values with Ba^2+^ were τ^o^ = 0.6 ± 0.1 ms (100%), τ^c^_1_ = 1.3 ± 0.1 ms (37%) and τ^c^_2_ = 23.0 ± 1.1 (63%). Values with Mg^2+^ were τ^o^ = 0.8 ± 0.1 ms (100%), τ^c^_1_ = 0.5 ± 0.1 ms (43%) and τ^c^_2_ = 11.2 ± 1.2 (57%).

It has been reported that RyR2 channel function can also be regulated by luminal Ca^2+^ [13], [14], [20], [25], [26]. Fewer studies have explored the sensitivity of these luminal sites to different divalent cations [13], [15]. Figure 2A shows RyR2 sensitivity to cytosolic Ca^2+^ with different luminal cations (Ca^2+^, Sr^2+^, Mg^2+^, Ba^2+^ and Cs^+^). In these experiments, the luminal cations were also the charge carriers (the current flows in lumen-to-cytosol direction). With luminal Cs^+^, Ca^2+^ or Sr^2+^, the channels were activated by cytosolic Ca^2+^ with an EC_50_ ∼ 3 µM (Fig. 2A). In contrast, significantly higher cytosolic Ca^2+^ levels were required to activate RyR2 in the presence of luminal Ba^2+^ or Mg^2+^ (EC_50_ ∼ 6.7 µM and 10.4 µM, respectively). At high cytosolic Ca^2+^ levels (>100 µM), the maximal P_o_ (plateau) value was similar in the presence of luminal Ca^2+^, Sr^2+^, Mg^2+^ or Ba^2+^. However, this value was significantly reduced (from P_o_ ∼0.8 to P_o_ ∼0.4) when we used Cs^+^ as charge carrier (luminal solution contained 100 mM Cs^+^ plus ∼500 µM luminal Ca^2+^). Open probabilities were much lower with luminal Cs^+^ solutions (where only micromolar contaminant Ca^2+^ was present) , but P_o_ at 0 mV cannot be accurately determined using this approach because the channels are prone to inactivation (results not shown). In Supporting Information (Fig. S1) we show that cytosolic Ca^2+^ increases P_o_ of RyR2 bathed with luminal Ca^2+^ by increasing the number of openings and by increasing event duration. A similar pattern (albeit with overal briefer events) is observed when RyR2 are bathed with luminal Sr^2+^, Mg^2+^ and Ba^2+^.

As shown in Fig. 2B, we then tested the effect of different luminal M^2+^ on the sensitivity of single RyR2 to caffeine. These experiments were conducted in the presence of 100 nM cytosolic Ca^2+^. As shown in the examples with luminal Ca^2+^ or Ba^2+^ (Fig. 2B, left and center panels), in the absence of caffeine (control conditions), the RyR2 had low P_o_. With luminal Ca^2+^ or Sr^2+^, caffeine activated RyR2 with similar EC_50_ values (Fig. 2B, right panel, open circles and open triangles). These caffeine-activated channels displayed long events (openings and closures), as shown in recordings (Fig. 2C, left top panels) and in dwell-time distribution histograms (Fig. 2C, right top panels). In contrast, when the current carrier was luminal Ba^2+^ or Mg^2+^, caffeine had little effect on RyR2, which were poorly activated or remained closed (Fig. 2B, right panel filled circles and triangles). In these conditions, most opening events were brief, as shown in recordings and dwell-time distributions (Fig. 2C bottom panels). A subsequent increase in cytosolic Ca^2+^ to 1 µM (Supporting Information, Fig. S2), activated these channels to high P_o_, suggesting they were still sensitive to caffeine. However, the length of openings did not reach values observed with caffeine and luminal Ca^2+^. In summary, caffeine enhanced the differential effects of luminal M^2+^ on RyR2 behavior. It is also apparent that the stabilization of RyR2 long openings, which has been associated with a conformational state denominated “high P_o_ gating mode” [27], [28], [29], requires luminal Ca^2+^. Indeed, in the absence of luminal Ca^2+^, RyR2 display a bursting behavior with alternating periods of low (flickering) and high P_o_, which is denominated modal gating [2], [28].

### Luminal M^2+^ affect RyR2 voltage-dependence

As shown in Fig. 2, there was a shift in the EC_50_ for cytosolic Ca^2+^ activation and the changes in RyR2 gating kinetics observed with different luminal M^2+^ (Fig. 2). This suggests either the presence of two different luminal sites (one for all M^2+^ and one selective for Ca^2+^ and Sr^2+^) or that luminal M^2+^ can affect cytosolic sites when flowing through the channel (feed-through) [4], [15], [17], [18], [19], [20]. If feed-through regulation exists, it could positively or negatively modulate RyR2 function. On one hand, luminal Ca^2+^or Sr^2+^ flowing through the channels could bind to the high affinity activating cytosolic M^2+^ binding sites and increase RyR2 activity. On the other hand, any M^2+^ coming from the lumen could interact with low affinity inhibitory cytosolic M^2+^ binding sites and decrease RyR2 activity. If feed-through produces further activation of RyR2, increasing SR membrane voltage (which increases the magnitude of lumen-to cytosol M^2+^ flux) should induce an increase in the P_o_ of RyR2 exposed to luminal Ca^2+^ or Sr^2+^ (i.e., voltage-dependence with a positive slope would be observed only with luminal Ca^2+^ or Sr^2+^). In contrast, if feed-through negatively modulates RyR2 function, the voltage-dependence curve for any M^2+^ should have a negative slope.

**Figure 3:**
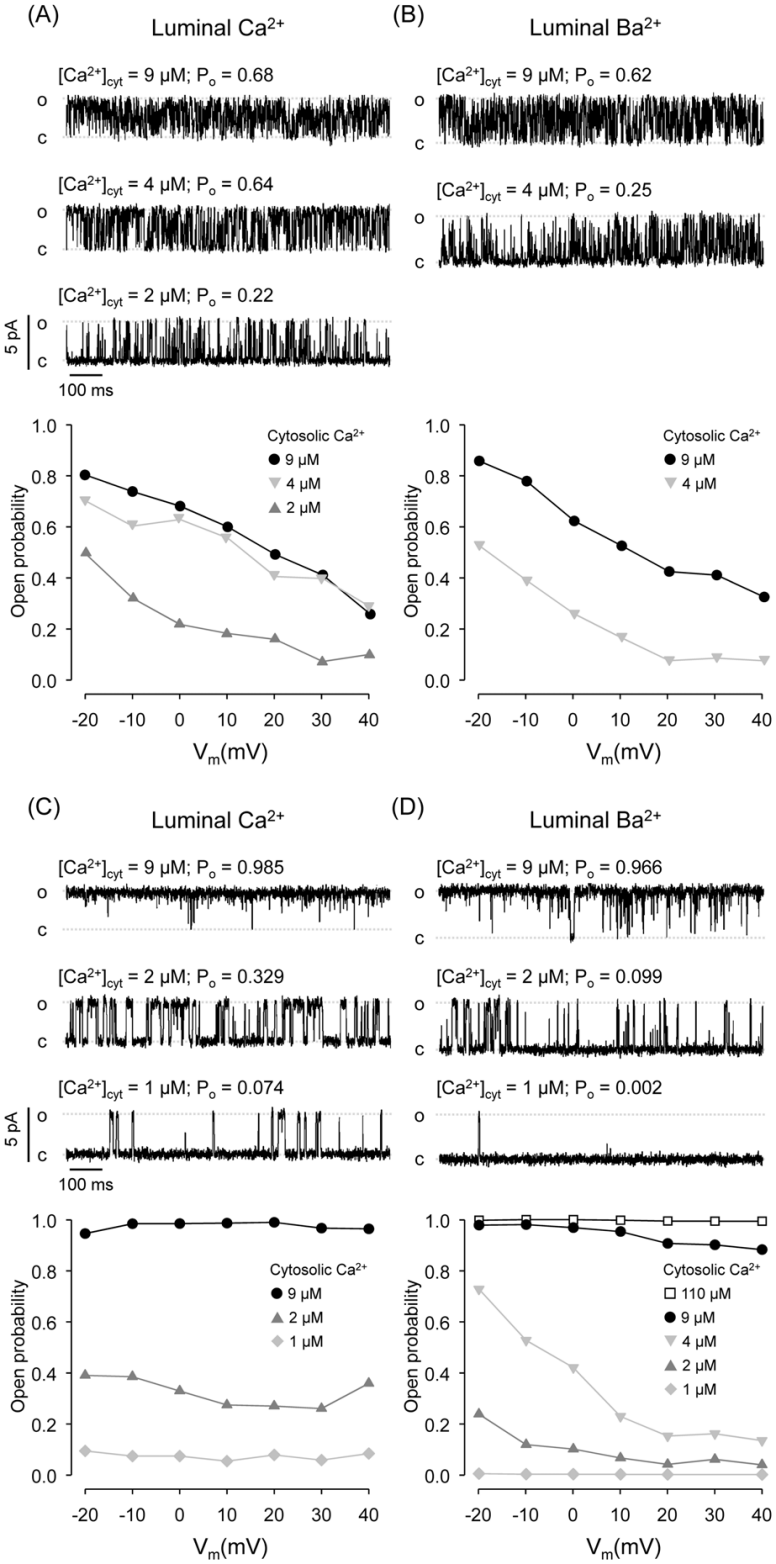
Effects of luminal M^**2+**^ on RyR2 voltage-dependence. (A-B) *Effect of luminal M^2+^ on cytosolic Ca^2+^ sensitivity and voltage dependence of a RyR2 that displays modal gating.* Single-channel recordings of a RyR2 exposed to different [Ca^2+^]_cyt_ with 50 mM luminal Ca^2+^ as current carrier (V_m_ = 0 mV). (B) Traces of the same channel shown in (A) after replacement of the luminal solution with 50 mM Ba^2+^. In (A) and (B), the bottom panels summarize the open probabilities as a function of holding voltage of the channel exposed to the indicated [Ca^2+^]_cyt_. Notice that increasing lumen → cytosol M^2+^ flux (by making V_m_ more positive) decreased P_o_ regardless of the identity of the luminal M^2+^. (C–D) *Effect of luminal M^2+^ on cytosolic Ca^2+^ sensitivity and voltage dependence of a RyR2 that displays high P_o_ mode.* Single-channel recordings of a RyR2 exposed to different [Ca^2+^]_cyt_ with 50 mM luminal Ca^2+^ *(C)* or 50 mM luminal Ba^2+^ *(D)* as current carrier (V_m_ = 0 mV). Bottom panels show open probabilities as a function of voltage. In contrast to the RyR2 shown in (A) and (B), this channel is virtually voltage insensitive when luminal Ca^2+^ is the current carrier. However, in the presence of [Ca^2+^]_cyt_≤4 µM and luminal Ba^2+^, increasing V_m_ decreased P_o_. This voltage sensitivity was abolished by further activating the channel upon addition of Ca^2+^ to the cytosol ([Ca^2+^]_cyt_ ≥9 µM).

We tested the effect of changing membrane voltage on RyR2 activity. Tested voltages ranged from −20 to +40 mV, which changed the magnitude of Ca^2+^ flux from ∼1 to 8 pA. As reported before for various aspects of channel function [10], [11], we found here that RyR2 are heterogeneous. Kinetic analysis of individual RyR2 was used to sort them into two groups: one displaying low-mid P_o_ mode with abundance of short lived gating events lasting from 1 to a few ms and the other with higher P_o_ and slower kinetics (long lasting events usually ranging from 10 to 100 ms). In Fig. 3, we show paired recordings performed on the same mid-low P_o_ mode RyR2 with either luminal Ca^2+^ (Fig. 3A) or luminal Ba^2+^ (Fig. 3B) as current carriers. We observed that increasing lumen-to-cytosol M^2+^ flux (by making V_m_ more positive) decreases P_o_ regardless of the identity of the luminal M^2+^. Notice, however, that P_o_ values at comparable cytosolic Ca^2+^ levels are higher with luminal Ca^2+^ than with luminal Ba^2+^. The differences in P_o_ correlate with more abundant and longer openings and shorter closed times in luminal Ca^2+^ versus Ba^2+^ (See Supporting Information, Fig. S3 and Table S1). For these voltage-dependent RyR2, we found that the probability to transition from closed to open (P_C→O_), estimated as number of openings divided by (1 – P_o_) recording time, also decreases with voltage but it is more marked with luminal Ba^2+^ (not shown).

Paired recordings showed in Fig 3C and 3D were taken from a single RyR2 displaying high P_o_ gating mode (slow kinetics). For this type of channel we detected little (if any) voltage-induced change in P_o_ when Ca^2+^ was the current carrier (Fig. 3C). However, when Ca^2+^ was replaced with Ba^2+^ (Fig. 3D) the channel displayed more frequent shorter events and definite voltage-dependence. Interestingly, voltage-dependence is not observed when the channels are fully activated by cytosolic Ca^2+^ (Fig. 3C and Fig. 3D). Indeed, full or partial suppression of voltage-dependence by increasing cytosolic Ca^2+^ levels was observed in most RyR2 (both populations). Increasing cytosolic Ca^2+^ levels from 2 µM to 4 µM in the presence of luminal Ba^2+^ increased P_o_ to values similar to those observed in the presence of luminal Ca^2+^ with 2 µM cytosolic Ca^2+^. However, openings are still shorter with luminal Ba^2+^ compared to luminal Ca^2+^ and the increase in P_o_ is mainly due to a shortening of closed events. Additionally, the decrease in P_o_ at more positive SR voltage observed with luminal Ba^2+^ results from a decrease in open times and an increase in closed times (See Supporting Information, Fig. S4 and Table S2). For this type of RyR2, the probability of transition from closed to open is voltage-independent with luminal Ca^2+^ but decreased with voltage with luminal Ba^2+^ (not shown).

### Replacement of 10% of luminal Ba^2+^ with Ca^2+^ suffices to match RyR2 behavior in 100% luminal Ca^2+^

**Figure 4:**
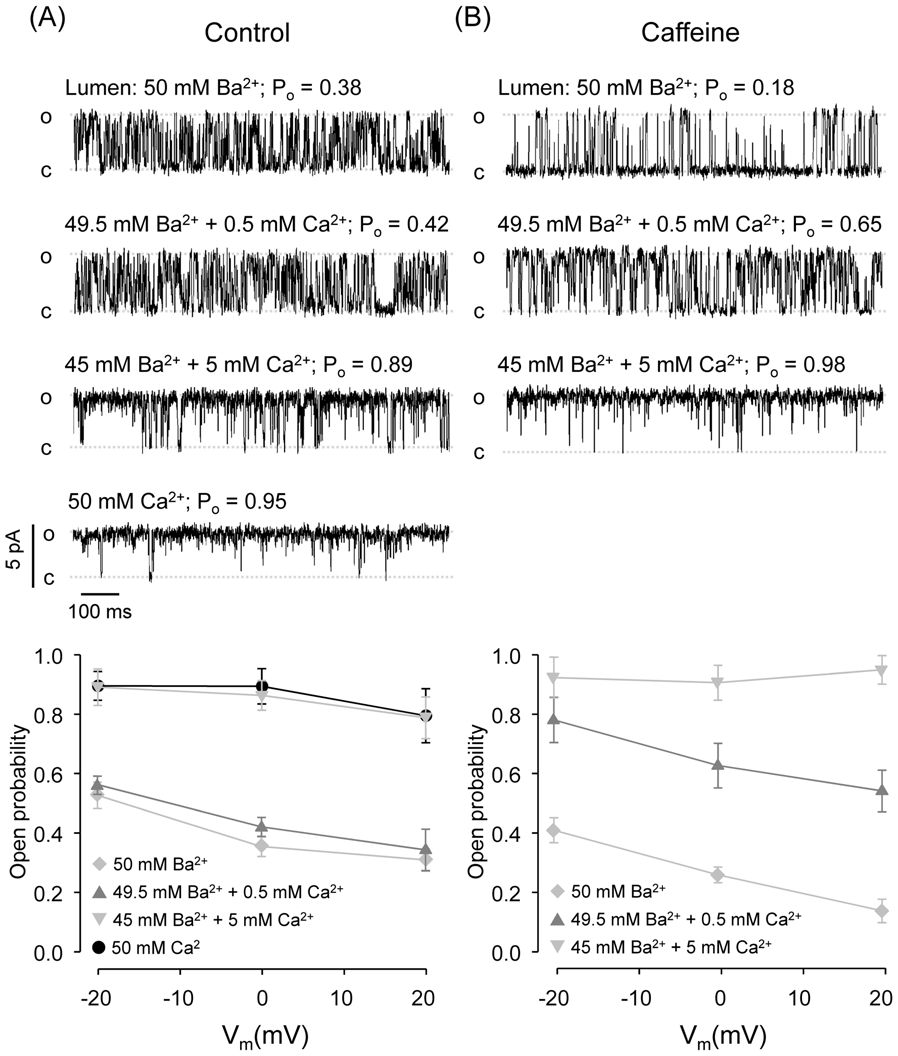
Effect of increasing luminal Ca^**2+**^ on the activity RyR2 bathed with luminal Ba^**2+**^ . **(A)** Single-channel recordings of a Ca^2+^-activated RyR2 ([Ca^2+^]_cyt_ = 4 µM) exposed to the indicated luminal M^2+^ mixtures (V_m_ = 0 mV). The bottom panel summarizes the open probabilities obtained at three different holding voltages. Notice that the channels display maximal activation and no voltage-sensitivity when luminal Ca^2+^ is higher than 5 mM. **(B)** Single-channel recordings of a caffeine-activated RyR2 ([Caffeine] = 20 mM; Ca^2+^ = 100 nM) exposed to the indicated luminal M^2+^ mixtures (V_m_ = 0 mV). As shown in the bottom panel, a small increase in luminal Ca^2+^ (0.5 mM) was enough to induce a significant increase in P_o_. Further increase in luminal Ca^2+^ (5 mM) caused maximal activation and removed voltage-sensitivity.

As mentioned above, RyR2 activity, gating kinetics and voltage-dependence varied according to the identity of the luminal M^2+^. Specifically, RyR2 display slower kinetics, higher P_o_ and less voltage-dependence with 50 mM luminal Ca^2+^ than with 50 mM luminal Ba^2+^. To test how much luminal Ca^2+^ is required to observe this behavior, we recorded partially activated (by 4 µM cytosolic Ca^2+^) RyR2 bathed with luminal 50 mM Ba^2+^ before and after adding increasing concentrations of Ca^2+^ to the luminal chamber. Subsequently, the luminal Ba^2+^ was completely replaced by 50 mM Ca^2+^. As shown in Fig. 4A, 5 mM Ca^2+^ suffices to increase P_o_ to values observed with 50 mM luminal Ca^2+^. As show in Fig. 4B, similar results were obtained when testing RyR2 partially activated by caffeine ([Caffeine] = 20 mM; [Ca^2+^ ]_cyt_ = 100 nM). Indeed, addition of 5 mM Ca^2+^ to the luminal chamber, increased the P_o_ to the same levels observed with 50 mM luminal Ca^2+^. Notice that 0.5 mM luminal Ca^2+^ induced a significant increase in the P_o_ of caffeine-activated channels while it did not affect Ca^2+^-activated channels. This would suggest that the interplay caffeine - luminal Ca^2+^ may produce a more robust change in RyR2 activity than luminal Ca^2+^ alone [24]. The main point to be taken from these experiments is that although the Ca^2+^ fluxes through the open RyR2 would be substantially different with luminal 5 mM Ca^2+^/45 mM Ba^2+^ versus 50 mM Ca^2+^ (as RyR2 Ca^2+^/Ba^2+^ permeability ratio is ∼1; [30]) there is no significant difference in RyR2 behavior between these conditions.

As shown in Fig. 4 caffeine-activated and Ca^2+^-activated RyR2 channels reached high P_o_ after luminal addition of 5 mM Ca^2+^, which could mask voltage-dependence. This lack of voltage-dependence was also observed in caffeine-activated RyR2 displaying partial activation (10 nM cytosolic Ca^2+^) with 50 mM luminal Ca^2+^ (Supporting Information, Fig. S5). This could reflect the fact that caffeine locks the channels in high P_o_ mode and RyR2 displaying this kind of gating are voltage-insensitive.

### Voltage-dependence does not seem to be related to luminal → cytosol M^2+^ flux

**Figure 5:**
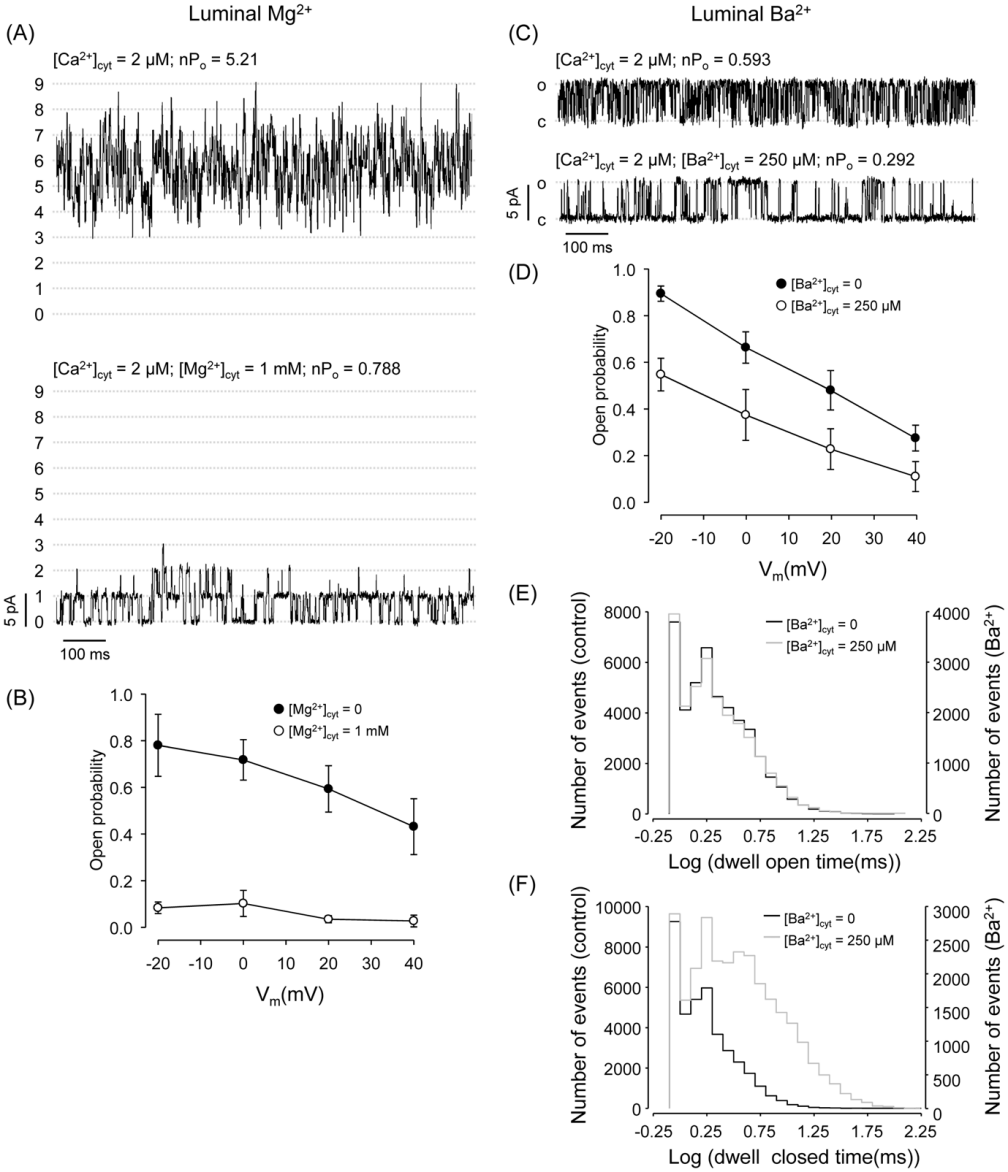
Effect of cytosolic Mg^**2+**^/Ba^**2+**^ on the behavior of RyR2 exposed to luminal Mg^**2+**^/Ba^**2+**^. (A) Multiple-channel recording of nine RyR2 exposed to 2 µM [Ca^2+^]_cyt_ with 50 mM luminal Mg^2+^ as current carrier (V_m_ = 0 mV) before (top) and after (bottom) addition of 1 mM cytosolic Mg^2+^. **(B)** Chart summarizing mean open probabilities (± S.E.M.) as a function of holding voltage of channels bathed with luminal Mg^2+^ under the indicated cytosolic conditions (n = 9 experiments). **(C)** Single-channel recording of a RyR2 exposed to 2 µM [Ca^2+^]_cyt_ with 50 mM luminal Ba^2+^ as current carrier (V_m_ = 0 mV) before (top) and after (bottom) addition of 250 µM cytosolic Ba^2+^. **(D)** Mean open probabilities (± S.E.M.) as a function of holding voltage of channels bathed with luminal Ba^2+^ before (filled circles) and after (open circles) addition of 250 µM cytosolic Ba^2+^ (n = 8 experiments). **(E)** Dwell open time distribution histograms of a single RyR2 exposed to luminal Ba^2+^ in the presence (grey outline) or absence (black outline) of 250 µM cytosolic Ba^2+^. Openings in the absence of cytosolic Ba^2+^ distributed with τ^o^_1_ = 1.61 ± 0.17 ms (85% events) and τ^o^_2_ = 6.18 ± 0.83 ms (15%). In the presence of cytosolic Ba^2+^, values were τ^o^_1_ = 1.09 ± 0.21 ms (70%) and τ^o^_2_ = 4.25 ± 0.36 ms (30%). **(F)** Dwell close time distribution histograms of RyR2 bathed with luminal Ba^2+^ before (black outline) and after (grey outline) addition of 250 µM cytosolic Ba^2+^. Closures in the absence of cytosolic Ba^2+^ distributed with τ^c^_1_ = 0.79 ± 0.24 ms (80% events) and τ^c^_2_ = 2.95 ± 0.57 ms (20%). In the presence of cytosolic Ba^2+^, values were τ^c^_1_ = 2.54 ± 0.12 ms (72%) and τ^c^_2_ = 13.37 ± 0.29 ms (28%)

The decreased sensitivity to cytosolic Ca^2+^ observed with luminal Ba^2+^/Mg^2+^ and the negative slope of RyR2 voltage-dependence could reflect the action of the M^2+^ feeding through the channel and producing: i) interference of flowing Ba^2+^/Mg^2+^ with cytosolic Ca^2+^-mediated activation; ii) interaction of flowing M^2+^ with cytosolic inhibitory low affinity M^2+^ binding sites or iii) a combination of both effects (only for luminal Ba^2+^/Mg^2+^). In any case, very high levels of the M^2+^ flowing through the channels should be reached at the cytosolic surface to produce inhibition. In previous experiments, we found that 1 mM cytosolic Mg^2+^ produces an increase in RyR2 EC_50_ for cytosolic Ca^2+^ from ∼2 µM to ∼10-20 µM [10], [11]. A similar EC_50_ for cytosolic Ca^2+^ was found when using luminal Mg^2+^ as charge carrier with no cytosolic Mg^2+^ added (EC_50_ ∼ 10 µM; Fig. 2A). If this effect results from luminal Mg^2+^ feeding through the channels we would expect the levels of Mg^2+^ reaching the cytosolic RyR2 surface to be around 1 mM. However, adding 1 mM Mg^2+^ to the cytosolic surface of channels bathed with 50 mM luminal Mg^2+^ produces a very large inhibition (Fig. 5A and B). This suggests that, if any, the levels of luminal Mg^2+^ reaching RyR2 cytosolic sites would be much less than 1 mM. Likewise, adding 250 µM cytosolic Ba^2+^ to RyR2 bathed with luminal Ba^2+^ induced a significant decrease in P_o_, without affecting voltage dependence (Fig. 5C and D). This again suggests that the levels of M^2+^ reaching cytosolic sites are lower than 250 µM.

An important observation in this paper is that luminal Ba^2+^ (or Mg^2+^) produce high frequency of short lived events, even when the channels are exposed to fully activating cytosolic Ca^2+^ levels (Fig. 2C). This could be attributed to Ba^2+^ (or Mg^2+^) feeding through the channel and transiently inhibiting RyR2 by binding to cytosolic sites [15], [17], [19]. This would be in agreement with Fig. 3 and Fig. 4 where we found large effects of voltage (or luminal to cytosol Ba^2+^ flux) on channel kinetics. However, it has been reported that in the absence of luminal Mg^2+^ (i.e., with luminal Ca^2+^ or Cs^+^), cytosolic Mg^2+^ increases the length of closures but does not significantly affect open times [12]. We have found similar results for the effects of both cytosolic Mg^2+^ and Ba^2+^ on channels bathed with luminal Ca^2+^ (results not shown). Indeed, in our paired experiments, the same RyR2 now bathed with luminal Ba^2+^, still displayed longer closures when exposed to 250 µM cytosolic Ba^2+^, but open event distribution did not change (Fig. 5E and 5F). Thus, previous studies and our current results suggest that the flickering observed in the presence of luminal Ba^2+^ (or Mg^2+^) would not be due to Ba^2+^ feed-through acting on a cytosolic site but it would be a luminal phenomenon.

Additional experiments were carried out with the purpose of preventing the putative effects of luminal M^2+^ reaching cytosolic binding sites. For that, we increased the buffering power of the cytosolic solution keeping [Ca^2+^]_cyt_ constant by adding mixtures containing Ca^2+^ and fast buffers (BAPTA/di-Bromo-BAPTA). If feed-through positively modulates RyR2 function, when using Ca^2+^ as charge carrier, higher levels of buffer would be expected to chelate the Ca^2+^ flowing from the lumen and decrease P_o_ by preventing Ca^2+^ interaction with cytosolic activating sites. Conversely, if inhibitory feed-through occurs, increasing buffering should only increase the activity of RyR2 exposed to luminal Ca^2+^ because BAPTA and di-Bromo-BAPTA are less effective as Ba^2+^ chelators and they do not significantly bind to Mg^2+^ [31]. Unexpectedly, increasing cytosolic buffer concentration induced a concentration-dependent increase in P_o_, regardless of the current carrier identity (Mg^2+^, Ca^2+^or Ba^2+^) (Results not shown). Some RyR2 activation is also found with EGTA or EDTA, which bind much more slowly to RyR2, suggesting that the buffering effect does not depend on the on-rate or affinity of the chelator for binding to the flowing M^2+^. This may suggests that BAPTA at high concentrations may have some Ca^2+^ buffering independent effects [32]. Another possibility is that contaminating transition metals, which may inhibit RyR2 activity, could be removed by high levels of BAPTA. Consequently, we used anions known to precipitate M^2+^, such as 50 mM sulfate (for Ba^2+^), or 10 mM fluoride (for Ca^2+^), as alternative chelators to sequester luminal M^2+^ flowing into the cytosol and prevent feed-through. However, we did not find significant changes in activity (negative results, not shown).

We tested the possibility that physiological monovalent ions (K^+^ and Na^+^), which are more electronegative than Tris^+^ or Cs^+^, could compete with M^2+^ flux effects in the RyR2 cytosolic surface or the vestibule. In this regard, it has been reported that K^+^ and Na^+^ may affect Ca^2+^ sensitivity of [^3^H]ryanodine binding [3], [8], [10], [33]. However, we and others found similar EC_50_ for Ca^2+^ using variable levels (100–250 mM) of different cytosolic cations such as Cs^+^ or Tris^+^ [1], [2], [4], [8], [10], [12]. Supporting these observations, we found that the P_o_ of RyR2 channels bathed with partially activating cytosolic Ca^2+^ (2 µM, near EC_50_ levels) or maximally activating cytosolic Ca^2+^ (10–100 µM) was not affected by Na^+^ or K^+^ (from 0 to 150 mM; n = 5 experiments each; negative results, not shown).

## Discussion

In this study, we found that at least four different types of binding sites are involved in the modulation of cardiac RyR2 by earth alkaline cations (M^2+^). On RyR2 cytosolic surface there are two different types of interacting M^2+^ binding sites: selective (Ca^2+^ > Sr^2+^) high affinity activating sites and nonselective (Ca^2+^ ∼ Mg^2+^ ∼ Sr^2+^ ∼ Ba^2+^) low affinity inhibitory sites. On the luminal surface of the channels, there are nonselective and Ca^2+^-selective binding sites (both activating and low affinity).

RyR2 were heterogeneous in their response to M^2+^. Heterogeneity included voltage-dependence, which varied according to the channel gating mode (RyR2 locked in high P_o_ mode did not display voltage-dependence). Luminal M^2+^ differentially affected the apparent affinity to Ca^2+^ of cytosolic sites ("trans effect"). This effect could be due to the ability of luminal Ca^2+^/Sr^2+^ to stabilize RyR2 high P_o_ gating mode (not found in presence of luminal Ba^2+^/Mg^2+^). Our results suggest that the differential effects of luminal M^2+^ mainly result from them acting in different luminal sites and would not be due to the luminal M^2+^ flowing through the pore and interacting with cytosolic binding sites.

### Cytosolic M^2+^ binding sites for RyR2 activation and inhibition

In the presence of 50 mM luminal Ca^2+^, both Ca^2+^ and Sr^2+^ bind to M^2+^ activating sites in the cytosolic surface of RyR and activate the channels in a cooperative fashion (n_H_ >1) to high P_o_ levels (>0.80) with different relative affinities (EC_50_ = 1.5 and 15 µM respectively). Selectivity of skeletal and cardiac RyRs for Ca^2+^ and Sr^2+^ as activators has been previously reported [4], [34], [35], however, there are discrepancies among these reports regarding the relative affinities for Ca^2+^ versus Sr^2+^. This could reflect differences between isoforms (RyR1 versus RyR2) and/or experimental conditions (e.g. presence/absence of Mg^2+^/ATP).

Mg^2+^ and Ba^2+^ do not induce channel openings, instead, they interfered with Ca^2+^ with Sr^2+^ activation. Many studies have previously defined the modulation of RyRs by Mg^2+^ [2], [4], [11], [12]. The novelty here is that Mg^2+^, the alkaline earth cation with the smallest radius (0.078 nm) and Ba^2+^ (the biggest one with 0.143 nm) bind to inhibitory cytosolic sites with equivalent affinity. The characteristics of selectivity usually described for M^2+^ binding sites [36] suggest that Ca^2+^ and Sr^2+^ (which have intermediate sizes, 0.105 and 0.127 nm respectively) would be able to bind to the same inhibitory sites. Mg^2+^ competition with Ca^2+^ binding to the activating cytosolic M^2+^ binding site has been proposed [1], [2], [3], [4], [11], [12]. In principle, the results presented in Fig. 1B and 1C would be in agreement with the hypothesis of competition for the same site, as Mg^2+^ and Ba^2+^ were more effective inhibitors at lower cytosolic Ca^2+^ concentrations. However, in the presence of caffeine, Ca^2+^ and Sr^2+^ activated RyRs more efficiently, while Mg^2+^ inhibitory efficiency was unchanged (Fig. 1D). This suggests that micromolar Ca^2+^ and Sr^2+^ bind to different cytosolic sites (referred as “Site A” [15]) than millimolar Mg^2+^ and Ba^2+^ (which bind to “Inhibitory M^2+^ Site 1”).

Our data also suggests that the low affinity inhibitory M^2+^ binding sites responsible for the bell-shaped response to cytosolic Ca^2+^ are non-selective (Fig. 1B). These sites have been proposed to be a different entity (putatively, “Inhibitory M^2+^ Site 2”) than the sites interfering with Ca^2+^ activation (Site 1) [15], [19]. As found here, these low affinity inhibitory M^2+^ Sites 1 and 2 cannot be distinguished based on M^2+^ selectivity. In our opinion, functional studies of RyR2 sensitivity to M^2+^ are also inconclusive. As shown here and as previously reported, occupation of Site 1 or 2 results in different kinetics [11], [12], [13]. Indeed, cytosolic Mg^2+^ and Ba^2+^ increase closed times of partially activated RyRs whereas addition of millimolar cytosolic M^2+^ correlates with a decrease in open times. In principle, these differences in response to M^2+^ may suggest two different cytosolic inhibitory M^2+^ sites. However, RyR2 gating status, which is heavily influenced by Ca^2+^ may also play a role [28], [29]. As we previously reported, low P_o_ mode is more sensitive to Mg^2+^ inhibition [10]. Thus, predominant inhibition of short events, which are more abundant at 5 µM cytosolic Ca^2+^, may mask flickery block during long openings. In contrast, at >100 µM cytosolic Ca^2+^, long openings are much more frequent [28] and the weight of the flickering block is much more manifest.

All partially activated RyR2 (cytosolic Ca^2+^<5 µM) are sensitive to inhibition by 1 mM Mg^2+^ [10], [12], while a significant fraction of fully activated RyR2 (cytosolic Ca^2+^ ∼ 200 µM) are not inhibited by increasing cytosolic Ca^2+^ to 5 mM [10], [37]. This could be indicative of different entities, with M^2+^ Site 1 present in all RyR2 and Site 2 present in ∼50% of the channels. Still, the observed differences may also reflect variability in RyR2 sensitivity to M^2+^. Indeed, we found that IC_50_ for RyR2 inhibition by Mg^2+^ (Site 1) range from 0.2 to 1 mM [11]. Thus, for Site 2, inhibition ranges may also be variable and much higher than 5–10 mM. Increasing cytosolic M^2+^ from 1 to 10 mM could generate additional unspecific electrostatic effects on the cytosolic RyR2 surface. Still, to confirm if the cytosolic inhibitory M^2+^ binding sites encompass one or more domains (Site 1 and Site 2) in the RyR2 molecule further molecular/structural data are required.

### RyR2 activation by luminal M^2+^

We found that the maximal Po reached by RyR2 in the presence of luminal M^2+^ is higher than that with luminal Cs^+^. Accordingly, it has been reported that there are Ca^2+^-sensing sites, accessible from the SR lumen, which participate in the regulation of RyRs [14], [15], [20], [25], [26]. Our data suggest that one type of luminal M^2+^ binding site, selective for Ca^2+^ and Sr^2+^, would affect RyR2 behavior. We also found that there are non-selective luminal M^2+^ binding sites where all M^2+^ (including Mg^2+^) may produce an increase in maximal Po. Calsequestrin (CSQ), a regulatory protein closely associated to RyR2, has been proposed to have a role in sensing luminal Ca^2+^ [1], [2], [3], [4], [38]. However, it has been suggested that luminal Ca^2+^ levels greater than 5 mM dissociate CSQ from RyR [38]. Since all of our channel reconstitutions were performed in the presence of 50 mM luminal Ca^2+^, RyR2-CSQ association may have been disrupted in our experiments. Thus, the luminal M^2+^ activation observed here would more likely involve M^2+^ binding sites located on the RyR2 protein itself.

In our hands, Sr^2+^ is the closest substitute for luminal Ca^2+^ (regarding the responses to cytosolic Ca^2+^ and caffeine). Notice, however, that saturating Sr^2+^ levels did not produce the same stabilization of high Po mode in caffeine-activated RyR2 as observed with luminal Ca^2+^ (Fig. 2C). Furthermore, the EC50 for cytosolic Sr^2+^ activation of RyR2 in the presence of luminal Ca^2+^ was less (∼ 20 µM) than that reported in previous studies for cytosolic Sr^2+^ activation in presence of luminal Sr^2+^ (EC50 ∼50 µM) [39]. Studies in cells also indicate that Ca^2+^ and Sr^2+^ differ in their ability to generate sparks [39].

Large differences in RyR2 behavior were found with luminal Ca^2+^ versus luminal Ba^2+^ or Mg^2+^. We estimate (paired comparisons) the probability of a RyR2 to open from the closed state (PC→O). At equivalent cytosolic Ca^2+^, PC→O was 2–20 times higher in RyR2 bathed with luminal Ca^2+^ versus luminal Ba^2+^ (the differences were more marked at positive SR holding voltages). This suggests that luminal Ba^2+^ decreases the on-rate and increases the off-rate of Ca^2+^ binding to activating cytosolic sites. A previous report found that at 0 mV, the effects of luminal Ba^2+^ on Po are only noticeable in caffeine-activated RyR2 [13]. However, in Ca^2+^-activated RyR2, equivalent Po (in the presence of luminal Ba^2+^ versus luminal Ca^2+^) was maintained by increasing the frequency of openings, which compensated the decrease in open times [13]. In our paired experiments, we only reached equivalent Po values by increasing cytosolic Ca^2+^ levels of RyR2 bathed with luminal Ba^2+^ (Supporting Information, Fig. S4 and Table S2). At equivalent Po values, the number of events with luminal Ba^2+^ was ∼5 times the number of events observed with luminal Ca^2+^. Three studies carried out in the presence of cytosolic ATP (1–2 mM) found that RyR function is largely affected by changes in luminal Ca^2+^ levels, but there were discrepancies regarding the sensitivity of luminal Ca^2+^ binding sites, with reported values ranging from micromolar to millimolar [14], [15], [16]. In the absence of ATP, increasing luminal Ca^2+^ to millimolar levels was reported to have no effect on maximal Po [14]. This is in disagreement with our results and a recent report [18] where high levels of luminal M^2+^ significantly increased maximal Po compared to that in the presence of luminal Cs^+^.

Using ATP-activated channels Laver et al. (2008) found that 50 µM Mg^2+^ decreases the activity of RyR2 both at negative (no significant Mg^2+^ flux) and positive SR voltages. The effects of luminal Mg^2+^ were attributed to competition with luminal Ca^2+^ sites and with cytosolic Ca^2+^ sites (feed-through modulation) [15]. However, other reports indicated that in absence of ATP, Mg^2+^ fluxes larger than 1 pA are required to observe voltage-dependence [17]. Different to our studies with saturating luminal Mg^2+^ and absence of ATP, these previous reports did not describe an increase in maximal Po compared with luminal Cs^+^ alone.

### Is RyR2 voltage-dependence a consequence of feed-through regulation?

Voltage dependence, observed with high flux of luminal divalent ions has been attributed to the interaction of the M^2+^ moving through the pore with activating and inhibitory sites in the cytosolic side of the channel [15], [17], [18], [19].

In our experiments we used saturated luminal M^2+^ which should maximize feed-through effects. All RyR2 bathed with luminal Ba^2+^ or Mg^2+^ display modal gating and exhibit voltage-dependence, even in the presence of caffeine. In contrast, when RyR2 are bathed with luminal Ca^2+^ a population displaying long openings (high P_o_ – slow kinetics gating mode) is not sensitive to voltage. Our results differ from a previous study where inhibitory effects of flux were found to be nearly equivalent with both Mg^2+^ and Ca^2+^ [18]. Indeed, in the presence of luminal Ca^2+^ only the subset displaying modal gating was voltage-dependent but addition of caffeine switched them to high P_o_ mode and abolished their voltage-dependence. These observations suggest that modal gating (ability to switch between high and low P_o_ modes) is required to observe voltage-dependence.

In the population of RyR2 where voltage dependence is observed, P_o_ decreased with increased M^2+^ flux (i.e., with increased SR positive voltage). It has been suggested that activating and/or inhibitory cytosolic Ca^2+^ binding sites of cardiac RyR2 and skeletal RyR1 can sense the Ca^2+^ flux through the open channel pore (feed-through M^2+^ regulation) [18], [19], [25]. Here we found that the EC_50_ for cytosolic Ca^2+^ is higher with luminal Mg^2+^/Ba^2+^ versus Ca^2+^/Sr^2+^ flowing through the channel (Fig. 2A). Previous studies suggested that if luminal Ba^2+^ or Mg^2+^ flowing through the pore reaches a concentration of ∼ 1 mM at the channel cytosolic surface, we should expect a change in the EC_50_ for cytosolic Ca^2+^ from 2 to ∼10–20 µM [11], [12], [13]. However, the data in Fig. 5 indicate that the amount of Ba^2+^ (or Mg^2+^) feeding through the channel would be much less than 0.25 mM, which could not explain the magnitude of the effect of luminal Ba^2+^ (or Mg^2+^) versus luminal Ca^2+^ (Fig. 2). Moreover, voltage-dependence of RyR2 bathed with luminal Ba^2+^ was not affected by the addition of cytosolic 0.25 mM Ba^2+^ (Fig. 5D), which is unexpected as cytosolic levels would be higher than those reached by the luminal Ba^2+^ feeding through the pore. In this regard, the percentage of inhibition of P_o_ by cytosolic Ba^2+^ at positive voltages is higher than that at negative voltages. However, higher fluxes of lumenal Ba^2+^ feeding through the pore at positive voltages should have better outcompeted cytosolic Ba^2+^ inhibitory effects. The additive effects of luminal Ba^2+^ with cytosolic Ba^2+^ may be a consequence of luminal Ba^2+^ promoting flickering (i.e. fast kinetics with short events and low P_o_ mode) and of cytosolic Ba^2+^ being more effective to block RyR displaying “low P_o_” mode [11]. Notice that “low P_o_” mode is promoted by positive voltages; i.e., by increased flux of luminal M^2+^ (See Figs. S3 and S4 and Tables S1 and S2).

Replacement of 5 mM of the luminal Mg^2+^ or Ba^2+^ with Ca^2+^ makes RyR2 behavior (activity, kinetics and voltage dependence) indistinguishable from that obtained with 100% (50 mM) luminal Ca^2+^. As RyR2 are equally permeable to all M^2+^ [2], [4], 5 mM Ca^2+^ would only account for 10% of the M^2+^ feeding through the pore, which may not explain the magnitude of the effect on channel properties. A possible explanation would be that 5 mM Ca^2+^ saturates the Ca^2+^-selective luminal M^2+^ binding sites, as suggested by previous studies [2], [14], [19].

Our experiments also support the idea of open RyR2 being insensitive to activation by Ca^2+^ feeding-through the channel as proposed by others in the absence of ATP and stimulating cofactors [19]. Although in this article we used fast chelating buffers (BAPTA and Bromo BAPTA), in a series of early experiments we compared RyR2 activity measured at 200 nM cytosolic Ca^2+^ using EGTA (n = 16) versus BAPTA/diBromoBAPTA (n = 37) and found no significant differences in P_o_ (0.014±0.004, n = 16 and 0.012±.026, n = 25). Analogous conclusions were obtained from similar experiments performed using even larger skeletal RyR1 populations (data not shown). If there were any activating effects of Ca^2+^ flux, we would expect large differences in P_o_ among experiments with different buffers, as it is estimated that Ca^2+^ levels at the cytosolic surface would reach much higher levels with EGTA (thousand times slower k_on_ for binding Ca^2+^) than with BAPTA [40]. Even with BAPTA, Ca^2+^ fluxes at positive voltages would have increased cytosolic [Ca^2+^] to micromolar levels [40]. This increase in cytosolic Ca^2+^ should have activated RyR2 (i.e., the slope of the voltage-dependence curve should be positive) and this effect should have been more evident for partially activated RyR2 (bathed with 100 nM Ca^2+^) in the presence of caffeine, where RyR2 sensitivity to cytosolic Ca^2+^ increases ∼20 times and full activation only requires cytosolic [Ca^2+^] ∼ 500 nM. As indicated, much higher levels of Ca^2+^ accumulate at the cytosolic surface of an open RyR2 with Ca^2+^ currents of up to 10 pA [40]. Our results suggest that activating effects of Ca^2+^ feeding through the pore may not be observed even in the presence of the stimulating cofactor caffeine.

As stated above, our results suggest that voltage-dependence requires modal gating. It is apparent that SR voltage depolarization destabilizes long openings and then short openings and long closures become more and more abundant. As shown, the effect of voltage can be counteracted in part by increasing cytosolic Ca^2+^ levels. This RyR2 behavior mirrors that of BK channels, which are also voltage- and Ca^2+^-gated channels [41]. Modal gating in K^+^ channels seems to result from dynamic interactions between various channel structures, including the pore helix, selectivity filter and external vestibule [42]. Similar mechanism could be in play for RyR2, which apparently have some structural homology with K^+^ channels [43], [44].

### Speculations on the role of Ca^2+^/Mg^2+^-mediated regulation of RyR2 function in cells

Our current and previously published data [10], [11], indicate that in cells, triggering of RyR2-mediated SR Ca^2+^ release by Ca^2+^ will depend on luminal and cytosolic resting levels of Ca^2+^ and Mg^2+^ as well as on the SR – cytosol membrane voltage. During the Ca^2+^ release event (usually generated by an array of RyR2 activating/deactivating in synchrony), luminal Ca^2+^ levels will decrease and cytosolic Ca^2+^ levels will increase. In contrast, cytosolic and luminal Mg^2+^ levels are expected to remain relatively constant (due to the Donnan effect of the polyanion CSQ, the SR luminal Mg^2+^ concentration would be expected to be similar or higher than that in the cytosol, which is ∼1 mM). Consequently, when intra-SR Ca^2+^ levels fall, Mg^2+^ would maintain occupancy of luminal non-selective M^2+^ binding sites and keep the channels active (at least partially) while cytosolic Ca^2+^ remain higher than 10 µM, which are the estimated Ca^2+^ levels on the RyR2 cytosolic surface [40], [45]. RyR2 activity could be reduced by a decrease in luminal Ca^2+^ levels, which in concomitance with a decrease in the driving force for Ca^2+^ flux, could greatly decrease SR Ca^2+^ release. On the other hand, the increase in cytosolic Ca^2+^ levels would tend to maintain RyR2 channels active even with lower levels of luminal Ca^2+^. How RyR2 close in cells for the termination of Ca^2+^ release is a process of still unknown nature [46].

RyR2 are known to alternate between two gating modes: low P_o_ mode and high P_o_ mode [27], [28]. According to our data, Ca^2+^-selective luminal sites may be important to stabilize RyR2 in high P_o_ mode. This suggests that a switch from high to low P_o_ mode may have physiological significance for RyR2 deactivation following SR Ca^2+^ depletion while the cytosolic Ca^2+^ levels remain elevated. Additionally, our results and previous reports suggest that open RyR2 do not sense the local increase in cytosolic Ca^2+^ they produce via luminal-to-cytosol flux. This may imply that if multiple RyR2 open simultaneously [47], [48] as during a Ca^2+^ spark [5] and they do not sense the released Ca^2+^ after activation, they might still be able to close in some synchrony, even when cytosolic Ca^2+^ levels remain high. For this, the RyR2 should remain refractory to activation by cytosolic Ca^2+^ for some time after they close (few milliseconds), as to allow for the dissipation of the local Ca^2+^ levels.

In conclusion, our studies indicate that the regulation of single RyR2 by M^2+^ (including physiological relevant ions such as Mg^2+^ and Ca^2+^) is a complex process which involves several interacting M^2+^ binding sites (luminal and cytosolic) that may dynamically modulate the function of heterogeneous RyR2 during local and global Ca^2+^ release events.

## Supporting Information

Figure S1 **Effect of cytosolic Ca^2^ on RyR2 kinetics.** **Top:** Open probability (P_o_, filled circles) and Probability of transition from close to open (P_C→O_, open circles) of RyR2 bathed with luminal Ca^2+^ (50 mM) as a function of cytosolic Ca^2+^. **Bottom:** Time constants for openings as a function of cytosolic Ca^2+^. Open time distributions were fitted with 2 components (τ_1_ and τ_2_).
(TIF)

Figure S2 **Effect of cytosolic Ca^2^ on RyR2 kinetics.** Representative traces of consecutive 4 minute-recordings (at 0 mV) of a RyR2 bathed with luminal Mg^2+^ (50 mM) under various conditions as indicated. Notice that in presence of caffeine, channels reach Po ∼0.5 with 0.3 µM cytosolic Ca^2+^ but openings are short.
(TIF)

Figure S3 **Effect of luminal Ca^**2+**^/Ba^**2+**^ on the kinetics of RyR2 exposed to the same cytosolic Ca^**2+**^ levels.** Dwell time distribution histograms for openings (left panels) and closures (right) on the same single RyR2 (paired measurements) in the presence of luminal Ca^2+^ (black outlines) or luminal Ba^2+^ (grey outlines). Cytosolic [Ca^2+^] was 4 µM. All four-minute recordings were taken at −20 mV (top panels), 0 mV (middle) and +20 mV (bottom).
(TIF)

Figure S4 **Effect of luminal Ca^**2+**^/Ba^**2+**^ on the kinetics of RyR2 displaying similar open probabilities.** Dwell time distribution histograms for openings (left panels) and closures (right) on the same single RyR2 (paired measurements) with luminal Ca^2+^ (black outlines) or luminal Ba^2+^ (grey outlines). Cytosolic [Ca^2+^] concentration was adjusted so that the open probabilities at 0 mV were similar in the presence of luminal Ca^2+^ compared with luminal Ba^2+^ ([Ca^2+^]_cyt_  = 2 µM and 4 µM with luminal Ca^2+^ and luminal Ba^2+^, respectively). All four-minute recordings were taken at the indicated holding voltages.
(TIF)

Figure S5 **Effect of holding voltage on caffeine-activated RyR2.** Mean open probabilities (±S.E.M.) as a function of holding voltage of RyR2 bathed with luminal Ca^2+^. All channels were tested in the presence of 20 mM caffeine and at the indicated cytosolic Ca^2+^ levels.
(TIF)

Table S1 Kinetic parameters calculated from the dwell time distribution histograms depicted in Supporting Information, Figure S3.
(DOCX)

Table S2 Kinetic parameters calculated from the dwell time distribution histograms depicted in Supporting Information, Figure S4.
(DOCX)
