# Supplementary material for: Modulation of Cardiac Ryanodine Receptor Channels by Alkaline Earth Cations
Source: PLoS One. 2011 Oct 21;6(10):e26693. doi: 10.1371/journal.pone.0026693 (PMC3198824; doi:10.1371/journal.pone.0026693)
Supplement: Table S1 — Kinetic parameters calculated from the dwell time distribution histograms depicted in Supporting Information, Figure S3. (DOCX) [file pone.0026693.s006.docx]

|  | **Luminal Ca^2+^** | **Luminal Ba^2+^** |
| --- | --- | --- |
| **-20 mV** | P_o_ = 0.702 | P_o_ = 0.523 |
|  | τ^o^_1_ = 2.50 ± 0.14 (81%) | τ^o^_1_ = 1.05 ± 0.19 (77%) |
|  | τ^o^_2_ = 8.71 ± 0.57 (19%) | τ^o^_2_ = 4.44 ± 0.46 (23%) |
|  | τ^c^_1_ = 0.53 ± 0.41 (67%) | τ^c^_1_ = 0.90 ± 0.25 (73%) |
|  | τ^c^_2_ = 1.97 ± 0.37 (33%) | τ^c^_2_ = 3.69 ± 0.45 (27%) |
| **0 mV** | P_o_ = 0.628 | P_o_ = 0.254 |
|  | τ^o^_1_ = 1.52 ± 0.21 (67%) | τ^o^_1_ = 0.80 ± 0.17(96%) |
|  | τ^o^_2_ = 5.22 ± 0.35 (33%) | τ^o^_2_ = 4.95 ± 2.16(4%) |
|  | τ^c^_1_ = 0.72 ± 0.24 (72%) | τ^c^_1_ = 1.66 ± 0.17(59%) |
|  | τ^c^_2_ = 2.90 ± 0.36 (28%) | τ^c^_2_ = 7.39 ± 0.21(41%) |
| **+20 mV** | P_o_ = 0.405 | P_o_ = 0.090 |
|  | τ^o^_1_ = 0.86 ± 0.28 (71%) | τ^o^_1_ = 0.55 ± 0.23 (95%) |
|  | τ^o^_2_ = 2.84 ± 0.48 (29%) | τ^o^_2_ = 2.65 ± 0.43 (5%) |
|  | τ^c^_1_ = 1.52 ± 0.18 (73%) | τ^c^_1_ = 4.79 ± 0.09 (70%) |
|  | τ^c^_2_ = 6.01 ± 0.40 (27%) | τ^c^_2_ = 26.06 ± 0.22 (30%) |
