# Supplementary material for: Modulation of Cardiac Ryanodine Receptor Channels by Alkaline Earth Cations
Source: PLoS One. 2011 Oct 21;6(10):e26693. doi: 10.1371/journal.pone.0026693 (PMC3198824; doi:10.1371/journal.pone.0026693)
Supplement: Table S2 — Kinetic parameters calculated from the dwell time distribution histograms depicted in Supporting Information, Figure S4. (DOCX) [file pone.0026693.s007.docx]

|  | **Luminal Ca^2+^** | **Luminal Ba^2+^** |
| --- | --- | --- |
| **-20 mV** | P_o_ = 0.391 | P_o_ = 0.726 |
|  | τ^o^_1_ = 5.91 ± 0.08 (71%) | τ^o^_1_ = 2.32 ± 0.23 (46%) |
|  | τ^o^_2_ = 25.21 ± 0.20 (28%) | τ^o^_2_ = 8.63 ± 0.18 (54%) |
|  | τ^c^_1_ = 4.67 ± 0.09 (50%) | τ^c^_1_ = 1.06 ± 0.20 (78%) |
|  | τ^c^_2_ = 31.51 ± 0.08 (50%) | τ^c^_2_ = 4.25 ± 0.53 (22%) |
| **0 mV** | P_o_ = 0.329 | P_o_ = 0.419 |
|  | τ^o^_1_ = 4.22 ± 0.09 (82%) | τ^o^_1_ = 1.30 ± 0.21 (61%) |
|  | τ^o^_2_ = 23.63 ± 0.37(18%) | τ^o^_2_ = 5.35 ± 0.25 (39%) |
|  | τ^c^_1_ = 4.38 ± 0.10 (59%) | τ^c^_1_ = 1.74 ± 0.16 (64%) |
|  | τ^c^_2_ = 33.13 ± 0.14 (41%) | τ^c^_2_ = 9.09 ± 0.24 (36%) |
| **+20 mV** | P_o_ = 0.270 | P_o_ = 0.151 |
|  | τ^o^_1_ = 3.54 ± 0.13 (66%) | τ^o^_1_ = 1.34 ± 0.11 (100%) |
|  | τ^o^_2_ = 13.25 ± 0.25 (34%) | τ^o^_2_ = n/a |
|  | τ^c^_1_ = 4.93 ± 0.11 (52%) | τ^c^_1_ = 3.69 ± 0.10 (65%) |
|  | τ^c^_2_ = 34.22 ± 0.12 (48%) | τ^c^_2_ = 18.84 ± 0.18 (35%) |
